# Video Game Addiction in Young People (8–18 Years Old) after the COVID-19 Pandemic: The Grey Area of Addiction and the Phenomenon of “Gaming Non-Pathological Abuse (GNPA)”

**DOI:** 10.3390/epidemiologia5030035

**Published:** 2024-08-12

**Authors:** Domenico Piccininno, Giulio Perrotta

**Affiliations:** 1 Department of Forensic Criminology, Forensic Science Academy (FSA), 84083 Castel San Giorgio, Italy; domenicopiccininno275@gmail.com; 2 Istituto per lo Studio delle Psicoterapie (ISP), 00185 Rome, Italy

**Keywords:** digital addiction, behavioral addiction, video game addiction, gaming disorder, gaming non-pathological abuse, videogames, maladjustment, deviance, antisociality, crime, criminality

## Abstract

Introduction: In the literature, video game addiction in youths is correlated with dysfunctional symptoms of anxiety, emotional disorders, and mood disorders, and the pandemic period of 2020–2022 has favored the aggravation of this behavioral addiction. Therefore, we identified the need to analyze this phenomenon with an emphasis on the risks and correlates related to deviance and maladjustment from a prospective perspective, seeking to understand the impact of the individual variables examined. Aim: To demonstrate whether the condition of “gaming non-pathological abuse” (GNPA) promotes psychopathological features of clinical interest, in the absence of a diagnosis of “gaming disorder” (GD). Materials and methods: A search performed on PubMed and administration of an ad hoc sociological questionnaire were used to investigate individual variables of criminological interest in a representative population sample (531 males/females, 8–18 years old, M: 14.4, SD: 2.5). Results: Statistical analysis showed that after the pandemic period, digital video game addiction was reinforced, feeding psychopathological traits consistent with anxiety, emotional disorders, and mood disorders. Variables correlated with impulsive, aggressive, and violent behavior related to age, gender, socio-environmental and economic background, and the severity of digital video game addiction. Conclusions: In the youth population (8–18 years), “gaming non-pathological abuse” (GNPA) is related to aggressive, impulsive and violent behaviors that foster phenomena of social maladjustment and deviance, especially in individuals living in disadvantaged or otherwise complex socio-economic and family contexts. Looking forward, the study of structural and functional personality profiles is essential in order to anticipate and reduce the future risk of psychopathological and criminal behavior.

## 1. Introduction

Video game use has constantly increased among children and adolescents, having uncertain consequences for their health [1]. Video game addiction or gaming disorder (GD) is defined as the constant and repetitive use of the Internet to play frequently with different players, potentially leading to negative consequences in many aspects of life. In clinical settings, addiction to video game use for recreational purposes is considered a psychopathology only if the behavioral pattern of persistent and recurrent video game use leads to significant impairment of daily functioning or psychological distress. It is diagnosed according to the Diagnostic Statistical Manual of Mental Disorders (DSM5-TR) criteria, Section 3, wherein over a period of 12 months at least, five criteria are enough confirm GD, with concerns resulting from gaming (cognitive salience), withdrawal symptoms when gaming is not possible, tolerance (need to increase gaming time), failure in attempts to control/reduce use, loss of interest in other hobbies or activities (behavioral salience), overuse despite acknowledging the existence of a problem, lies about time spent playing, video games use to sedate/regulate/reduce an unpleasant emotional experience, loss or impairment of relevant interpersonal relationships, and impairment of school or work performance [2,3]. As recent technological development has allowed easy access to gaming on many devices, video game addiction has become a serious public health problem with increasing prevalence [4]. The non-pathological abuse of video games for recreational activity (“gaming non-pathological abuse”, or GNPA), which does not meet the diagnostic criteria of the DSM-5-TR, is not yet considered in the scientific literature, although it may be a potential cause of psychological distress for the subject [5,6,7].

With the advent of the global COVID-19 pandemic, social isolation and fear of viral contagion resulted in profound changes in social relationships among people [8], generating or fueling the psychopathological symptoms of anxiety, emotional distress, low mood [9,10,11], and psychosis [12] in those affected by internet gaming disorder and video game addiction. It also fostered deviant and criminal conduct, such as cyberbullying [13] and other behavioral addictions [2,6,7,14,15,16,17,18,19] that promote isolation, aggression, and deviant and criminal behavior and suicide risk [6,7,20,21,22] and also influence cognitive and behavioral performance [23]; thus, all of the above are clinically relevant conditions worthy of psychotherapeutic [24] and pharmacological evaluation and treatment [25,26].

Recent systematic review and meta-analysis studies have shown and confirmed that the prolonged effects of the recreational use of video games determine an increase in anxiety (phobias, fixations, panic and sleep disorders), emotional (aggression and impulsivity) and mood (manic and depressive) symptoms, and risk of suicidal ideations [27,28,29,30]. Additionally, from a neurobiological point of view, recent studies have shown that the reckless use of certain types of games, violent ones in particular, can impact brain circuits by leading to their structural and functional modification of the cognitive performance of attentional, control–rational, and visuospatial skills and reward processing [31,32]. However, contrary studies also emerge from the literature, praising the cognitive, motivational, emotional, and social benefits of the playful activity of using video games [33,34,35] in terms of the cognitive behavioral approach to therapy [36] and cognitive and psychopathological assessment [37,38,39,40], thus leading to confusion between the outcomes of published research.

The purpose of the present study is to analyze the impact of the post-COVID-19-pandemic period on the youth population (8–18 years old) who use video games for recreational purposes, in the absence of a psychopathological diagnosis of “Gaming Disorder” (GD) but with an attenuated condition that the literature does not take into consideration (i.e., “gaming non-pathological abuse”, or GNPA) and then correlate the data obtained from the administration of a questionnaire survey to assess the severity of dependency using the variables of age gender; socio-environmental and economic background; and impulsive, aggressive, and violent behaviors, if present. In the absence of a GD diagnosis according to DSM-5-TR criteria, the aim was to demonstrate that such a condition is still worthy of socio-clinical intervention because it can generate potential psychological distress.

## 2. Materials and Methods

### 2.1. Materials and Methods

**Figure 1:**
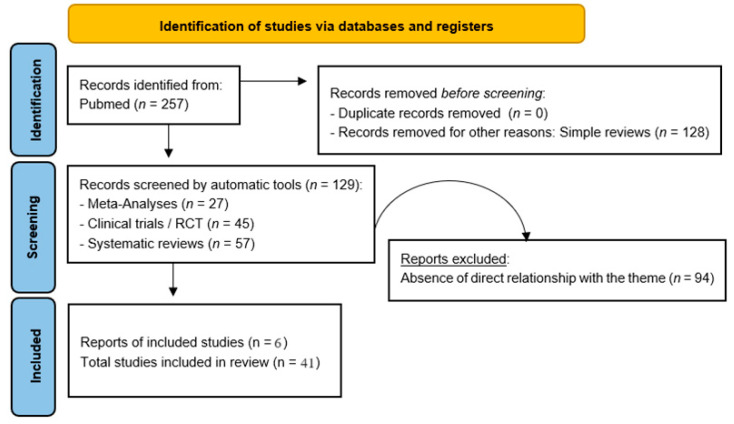
PRISMA flow diagram template. Adapted from Page M.J. et al. [41].

From January 1991 to June 2024, we actively searched PubMed for systematic reviews, meta-analyses, clinical trials, and randomized controlled trials using “Addiction AND Video games”, selecting 257 eligible results, 35 of which were included by removing duplicate content, irrelevant items, and absence of search items. To get a broader and more complete overview of the topic, 6 more references were added (from elsewhere and not the Pubmed platform, being materials from the academic literature), for a total of 41 results. Simple reviews, opinion contributions, or publications in popular volumes were excluded as redundant or not relevant to this work. An artificial intelligence program was not used for the automatic selection of results, but the tools and services offered by the PubMed platform were used. The search was not limited to English and Italian language papers (Figure 1).

The use of the literature was necessary to construct the introduction section of this study and to understand the usefulness of delving into the topic of “gaming non-pathological abuse” (GNPA), which is still not discussed in the literature because it is not considered clinically relevant.

Having identified the criteria for selecting the population, various meetings in educational institutions were arranged to raise awareness among students and ask for their participation, subject to prior signing of informed consent and data processing, signed by parents or by students over 18. Having indicated the link to fill out the specially prepared Sociological Questionnaire (with 50 items, analyzing the variables listed in Table 1, with single yes/no, multiple, or open-ended responses), the students (independently or helped by a familiar adult) logged on to complete it. The present research work was carried out from January 2023 to June 2024. All participants were guaranteed anonymity and the ethical requirements of the Declaration of Helsinki were met. Since this research was not funded by anyone, it is free of conflicts of interest.

Data from the questionnaires were tabulated in an Excel database, and statistical analysis was performed using SPSS software, version 27. Descriptive analyses of categorical and continuous variables and an exploratory analysis were conducted to identify potential associated factors; Chi-square tests were performed at a 5% significance level to identify the associated dependent and independent variables. An exploratory bivariate and multivariate analysis was then conducted to determine the value of the selected variables. Multivariate logistic regression analysis was performed with a 95% confidence interval based on the variables selected from the bivariate model with a significance level of up to 20%. Variables with collinearity (≥5.0) were excluded to improve the predictive value of the multivariate analysis.

### 2.2. Setting and Participants

The clinical population of the study consists of children and adolescents aged 8 to 18 years, either exposed or not to COVID-19 infection, who attended elementary, middle and high schools over three years, from 2020 to 2022. Inclusion criteria are related to the participant’s age at the time of enrollment to the present study, declaration of video game use for leisure (for at least 1 h a day), absence of GD diagnosis according to DSM-5-TR criteria, good health, Italian nationality, defined sexual gender (male/female), and informed consent and data processing, personally signed if 18 years old or signed by parents/guardian if a minor. Exclusion criteria relate to age under 8 years or over 18 years at the time of enrollment in the present study, declaration of non-use of video games for leisure or in any case for a daily use of less than 1 h, presence of one or more pathological conditions both physically and mentally certified, foreign nationality, undefined sexual gender (transgender), and absence, incorrect or withdrawal of informed consent and data processing. The control population of the study consists of children and adolescents aged 8–18 years, exposed or not to COVID-19 infection, who were attending elementary, middle and high schools over three years (2020–2022). Inclusion criteria are related to the participant’s age at the time of enrollment to the present study, declaration of video game use for leisure for less than 1 h, absence of GD diagnosis according to DSM-5-TR criteria, good health, Italian nationality, defined sexual gender (male/female), and informed consent and data processing signed personally if 18 years or by parents or legal representatives if under age. Exclusion criteria include participants of less than 8 years or older than 18 years at the time of enrollment in this study, declaration of video game use for leisure or more than 1 h per day, presence of one or more pathological certified conditions, both physical and mental, foreign nationality, undefined sexual gender (transgender), and absence, erroneousness or withdrawal of informed consent and data processing.

The schools were selected randomly but considering geographical calibration to cover all territorial areas of the country (Italy) for an equal sample distribution. In total, 9 schools participated in the study: 3 from northern Italy, 3 in central Italy, and 3 in southern Italy. We identified the sample size with the minimum *n* through a statistic calculation of the total interviewable students and daily use of video games for recreational purposes, determining a power of 80% and a significance level of 95%, with a design error of 5%. Therefore, a minimum required sample of at least 520 school-age children was identified. The drop-out rate was 0/531 (0.0%).

## 3. Results

### 3.1. Descriptive Statistics

The selected clinical population group (participants who reported daily playful activity using video games for over 60 min) is made up of 271 participants (clinical patients), ambisexual, aged 8–18 years (M: 14.3, SD: 2.5), while the control group (participants who reported daily playful activity using video games for a maximum of 60 min) is made up of 260 participants, ambisexual, aged 8–18 years (M: 14.5, SD: 2.5). The total population consists of 531 participants, ambisexual, aged 8–18 years (M: 14.4, SD: 2.5) (Table 2).

Statistical findings regarding the variables of clinical interest are shown in Table 3.

### 3.2. T-Test for Independent Samples

We performed a T-test for the selected clinical variables using independent samples, designating “3” as the dependent variable; significant results emerged, as shown in Table 4.

### 3.3. Statistical Trends

**Figure 2:**
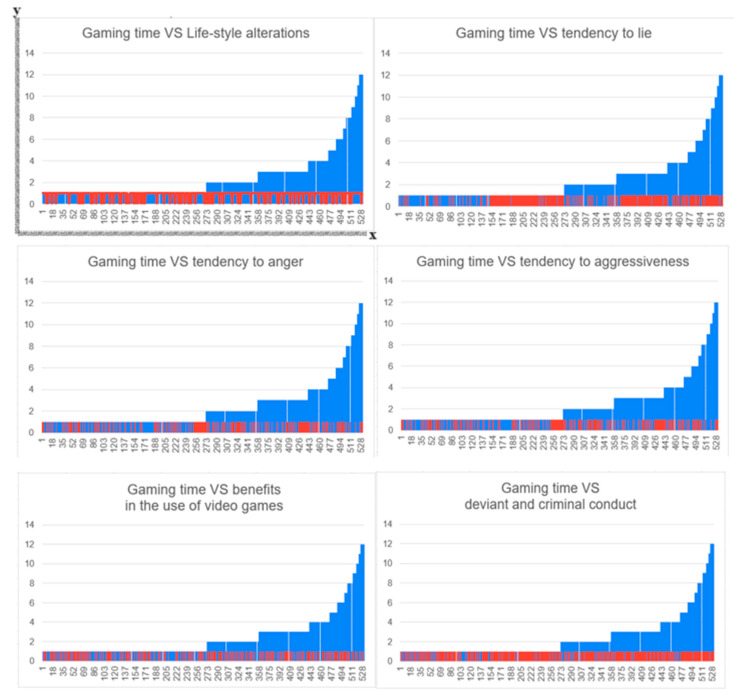
Graph of growth curves between the variable representing the daily duration of gaming time and the tendency to lie, feel anger, show aggression, perceive benefits from gaming activity, and engage in deviant and criminal behavior. The X-axis shows all study participants according to an increasing scale of severity (concerning the number of hours devoted to playful activity with video games; the more hours devoted, the more severe the need to use video games as an addictive tool), while the Y-axis shows hours of video game use. The subjects in the clinical group are in blue, while the subjects in the control group are in red.

The variables of clinical interest are grouped into 6 groups: (a) “time management” group (4, 5, 6); (b) “loss of time” group (7, 8, 9, 10, 11); (c) “reason for addiction” group (12, 13, 14, 15); (d) “subjective perception” group (16, 17, 21, 22); (e) “deviant and criminal conduct” group (18, 19, 20, 26); and the (f) “online mode” group (23, 24, 25). From the analysis of the data, upon carrying out the frequency assessment, a constant and directly proportional trend emerged between the duration of hourly playing time (or gaming time) and variables 16, 18, 19, 20, 21, and 26 (Figure 2).

### 3.4. Binary Correlations

A binary correlation between the generic and clinical variables was performed, detecting a range of significance values concentrated in all subgroups of interest (Table 5).

## 4. Discussion

The analyzed data from the selected population sample allowed for detailed descriptive analysis and correlations between variables. In particular, from the overall data obtained, it is possible to make the following claims:

1. The variable for the daily hourly duration of video game play activity (4) is inversely proportional to the number of participants in the present study; within 12 h, 440/531 (82.9%) subjects play for less than 3 h per day, and as the daily hours increase, the number of participants decreases. The 1 h cut-off was determined based on the scientific literature referenced herein, which considers video game use of less than 60 min as non-pathological.
2. In the clinical subgroup “time management” (4, 5, 6), variable 5 is represented by 6/10 of the overall sample, demonstrating that the participants frequently use some evening and nighttime hours, which are normally devoted to sleep activity, to play. These data are consistent with the result of variable 7, which is represented by only 10% of the research population. Variable 6 accounts for just under 1/3 of the sample, showing that more than 2/3 of the video game activity is a game shared with other people, consistent with variable 23 of online gaming, which is represented by almost 4/5 of the total population sample.
3. In the “loss of time” clinical subgroup (7, 8, 9, 10, 11), the variable most represented is #11, showing that time spent on gaming absorbs that to be spent on food and eating in almost 6/10 of the samples. This is followed by variable 8, with almost 1/3, showing that video game play activity is closely related to age (*p* < 0.001).
4. In the clinical subgroup “reason for addiction” (12, 13, 14, 15), variables indicating school and family difficulties (15) and the absence of friends (12) are more represented, by more than 8/10 for the former and a little more than 1/4 for the latter. This confirms that the ludic activity of video games can be considered an “emotional refuge” for pre-adolescents and adolescents.
5. In the clinical subgroup “subjective perception” (16, 17, 21, 22), more than 50% of the population sample states that they perceive alterations in the normal conduct of life due to video games (variable 16), as the time devoted to gaming must be taken away from other activities; however, this finding must be compared with the outcome of variable 21, which confirms the positive perceptions of the participants in more than 6/10 of the total population sample: these data must be read with caution, as a positively perceived state does not necessarily correspond to an actual state of well-being, as evidenced by the variables of the deviant and criminal conduct clinical subgroup. Therefore, this apparent state of well-being is likely linked to the reward circuit arising from video game use, which is known to be involved during these activities.
6. Variable 17 confirms that the pandemic period exacerbated video game addiction, both during and after the conclusion of the pandemic, in both cases for more than 6/10 of the total population sample. This variable correlated with both age (R = 0.198, *p* < 0.001) and longer duration of video game use (R = 0.102, *p* = 0.019) but also with time taken away from study (R = 0.139, *p* = 0.001), time takenn away from friends (R = −0.198, *p* < 0.001), use of lies (R = 0.130, *p* = 0.003), anger (R = 0.197, *p* < 0.001), aggression (R = 0.199, *p* < 0.001), and preference for video games with violent themes (R = 0.155, *p* < 0.001).
7. In the “deviant and criminal conduct” clinical subgroup (18, 19, 20, 26), 40–50% of participants reported feeling anger and aggression and using lying as a strategy for not taking responsibility.
8. A worrying result emerges from variable 22, in which more than 8/10 of the sample population prefers video games with violent and/or aggressive contents, as if the individual tends to sublimate unconscious destructive energy through gaming or as if the more one releases emotional and nervous tension while playing video games, the greater the urge to continue, thus reinforcing a confirmatory pattern. The latter interpretation would seem to be confirmed by the bivariate correlation analysis between this variable and variables 12 (R = 0.123, *p* = 0. 005), 15 (R = 0.111, *p* = 0.010), 16 (R = 0.115, *p* = 0.008), 17 (R = 0.155, *p* < 0.001), 18 (R = 0.089, *p* = 0.041), 19 (R = 0.651, *p* < 0.001), and 20 (R = 0.640, *p* < 0.001).
9. Finally, in the clinical subgroup “online mode”, alarming data emerge from pre-adolescent and adolescent subjects who play online video games without parental control, who find themselves pulled into a virtual reality where almost 8/10 of the research sample experience inappropriate contact with adults, and the youngest play violent games, resulting in direct and indirect participation in deviant and criminal conduct.
10. Frequent use beyond 2 h a day of playful activity with violent-type video games increases aggressive, violent, and manipulative behavior through lying, facilitating the process of imitating deviant and criminal behavior in real life. Age is directly correlated with deviant and criminal conduct, both of others (R = 0.233, *p* < 0.001) and one’s own (R = 0.266, *p* < 0.001), while the greater the use of video games for recreational purposes in terms of time, the more frequent the presence of manipulative (R = 0.123, *p* = 0.005), angry (R = 0.094, *p* = 0.030), and aggressive (R = 0.090, *p* = 0.037) behaviors.

The value of the new nosographic category “gaming non-pathological abuse” (GNPA), in our opinion, has the same clinical importance as the better-known “gaming disorder” (GD) because in this study, we have shown that in the absence of positivity for at least five criteria, subjects who nevertheless use video games for recreational purposes for more than 1 h per day present a marked increase in values related to aggressive, manipulative and angry conduct, and these underlie deviant and/or criminal attitudes in developmental age. The absence of positivity for at least five criteria does not characterize the diagnosis of GD but is certainly worthy of clinical evaluation if the temporal use of the ludic tool is more than 1 h per day, regardless of gender and mode of use, so that we might prevent or intervene promptly in attitudes that may already be deviant or criminal (or become so if reinforced).

### Limitations, Implications for Clinical Practice, and Prospects

A set of variables of socio-criminological and clinical nature were used in the present study, but they do not entirely represent the phenomenon, because it was not possible to perform a regression and thus obtain statistically significant values. However, thanks to the good results obtained, we can reflect upon the need to organize a larger population sample and a set of variables that also include health data, outcomes of test admini-stration, and the reconstruction of personality profiles. This study must be considered the first piece of a larger research work that hopefully can be more broadly developed as new means are attained, including economic ones. Lastly, the survey instrument used is not clinical but socio-criminological, as the goal of this research was not to diagnose the selected population sample but to understand the direct and indirect consequences of video game overuse. For this reason, a questionnaire with survey functions was used rather than a standardized and validated test.

## 5. Conclusions

A large sample of the youth population (8–18 years old) was studied to demonstrate whether addictive behaviors in the use of video games for leisure, which fail to meet the diagnostic criteria for Gaming Disorder (GD), are worthy of investigation as a potential threat to the subject’s psychological stability. The study shows that “gaming non-pathological abuse” (GNPA), understood as an attenuated form of GD that has fewer than five diagnostic criteria required for nosographic diagnosis, is also related to aggressive, impulsive, and violent behaviors that foster phenomena of social maladjustment and deviance, especially in individuals living in already disadvantaged and/or complex socio-economic and family contexts. The assessment of socio-environmental, family and personal variables—such as childhood trauma, poverty, degrading social context, and highly deviant or criminal friendships—together with the study of structural and functional personality profiles is essential in investigating this phenomenon from a clinical point of view, in the absence of a GD diagnosis, to anticipate and/or reduce the future risk of psychopathological and criminal behavior.

## Figures and Tables

**Table 1:** List of selected variables, specifying type and description.

N	Variable	Type_Variable	Type_Answer	Description
1	Age	Generic	Continued: 8–18 years old	Variable related to age
2	Gender	Generic	Dichotomous_Yes/No	Variable related to gender
3	Cluster	Generic	Dichotomous_Yes/No	Variable related to membership in the clinical group (daily recreational use of video games, more than 60 min) or control group (daily recreational use of video games, less than or equal to 60 min), including non-continuous
4	Time spent per day on recreational activities such as video games	Clinic	Continued_1–12 h	Variable related to the specific daily time (expressed in h/hour) spent on video game playing activity
5	Night game	Clinic	Dichotomous_Yes/No	Variable related to the time spent playing video game during nighttime hours that are usually devoted to sleep and rest
6	Solo game	Clinic	Dichotomous_Yes/No	Variable related (most of the time) to the performance of the video game as a playful activity in solitude rather than in company or sharing
7	Time taken away from study	Clinic	Dichotomous_Yes/No	Variable related to the time taken away from studying in favor of video games
8	Time taken away from friends	Clinic	Dichotomous_Yes/No	Variable related to the time taken away from friends in favor of video games
9	Time taken away from going out	Clinic	Dichotomous_Yes/No	Variable related to the time taken away from going out in public and attending events in favor of videogames
10	Time taken away from personal hygiene	Clinic	Dichotomous_Yes/No	Variable related to the time taken away from personal hygiene in favor of videogames
11	Time taken away from eating	Clinic	Dichotomous_Yes/No	Variable related to the time taken away from eating in favor of video games
12	Reason for addiction: absence of friends	Clinic	Dichotomous_Yes/No	Variable related to the reason for time spent on video game playing activity (absence of friends)
13	Reason for addiction: reduced interest in the outdoors	Clinic	Dichotomous_Yes/No	Variable related to the reason that justifies the time devoted to the video game playing activity (reduced interest in spending time outdoors, in public, or on group activities)
14	Reason justifying addiction: poor school performance	Clinic	Dichotomous_Yes/No	Variable related to the reason that justifies the time spent on video game playing activity (poor school performance)
15	Reason justifying addiction: emotional and family difficulties	Clinic	Dichotomous_Yes/No	Variable related to the reason for time spent on video games playing activity (emotional and family difficulties)
16	Alterations in life conduction as a result of video game-like activity	Clinic	Dichotomous_Yes/No	Variable related to the perceived negative effect on one’s lifestyle, resulting from the performance of playful activities with video games
17	Reinforcement of video game addiction in the pandemic	Clinic	Dichotomous_Yes/No	Variable related to the perceived temporal increase in playful activities with video games during and after the pandemic period
18	Use of lies	Clinic	Dichotomous_Yes/No	Variable related to telling lies during and after playing video games (e.g., to continuing to play or not taking specific responsibility)
19	Anger	Clinic	Dichotomous_Yes/No	Variable related to the expression of anger during and after the performance of playful activities with video games (e.g., to continuing playing or not taking specific responsibility)
20	Aggressiveness	Clinic	Dichotomous_Yes/No	Variable related to the display of aggressivity during and after the performance of playful activities with video games (e.g., continuing playing or not taking specific responsibility)
21	Subjective perception of benefits of using video games	Clinic	Dichotomous_Yes/No	Variable related to the subjective perception of possible benefits from performing playful activities with video games
22	Preferred game setting (violence)	Clinic	Dichotomous_Yes/No	Variable related to the preference for a certain video game types (violent type/non-violent type)
23	Use of video games in online mode	Clinic	Dichotomous_Yes/No	Variable related to the prevalence or not of using video games in on-line or off-line mode
24	Online encounters with individuals younger than the recommended age	Clinic	Dichotomous_Yes/No	Variable related to online meetings with individuals younger than the recommended age (e.g., 18 years old for violent games)
25	Witnessing others of deviant or criminal conduct	Clinic	Dichotomous_Yes/No	Variable related to having witnessed online deviant or criminal conduct by other users
26	Simulation or implementation of deviant or criminal conduct	Clinic	Dichotomous_Yes/No	Variable related to having witnessed or simulated online deviant or criminal conduct seen in video games

**Table 2:** Population group (*n*_%).

Clinical Group			
Age_y_Range	Male_*n* (%)	Female_*n* (%)	Total_*n* (%)
8–12	36 (29.7%)	46 (30.7%)	82 (30.2%)
13–18	85 (70.3%)	104 (69.3%)	189 (69.8%)
**Total**	121 (44.6%)	150 (55.3%)	**271** (100%)
**Control group**			
**Age_y_range**	**Male_*n* (%)**	**Female_*n* (%)**	**Total_*n* (%)**
8–12	53 (30.4%)	19 (22.1%)	72 (27.7%)
13–18	121 (69.6%)	67 (77.9%)	188 (72.3%)
**Total**	174 (66.9%)	86 (33.1%)	**260** (100%)
**Total group**			
**Age_y_range**	**Male_*n* (%)**	**Female_*n* (%)**	**Total_*n* (%)**
8–12	89 (30.2%)	65 (27.5%)	154 (29.0%)
13–18	206 (59.8%)	171 (72.5%)	377 (71.0%)
**Total**	295 (55.5%)	236 (45.5%)	**531** (100%)

**Table 3:** Variables of clinical interest—statistical findings.

N_Variable	Nomenclature	H/n_Case			% Total
		H_Time	N_Case_Total	N_Case_Clinic	
4	Time spent per day on recreational activities such as video games	1	271	11	51.0
		2	85	85	16.0
		3	84	84	15.8
		4	33	33	6.2
		5	13	13	2.4
		6	12	12	2.3
		7	5	5	0.9
		8	8	8	1.5
		9	6	6	1.1
		10	4	4	0.8
		11	3	3	0.6
		12	7	7	1.3
**N_Variable**	**Nomenclature**	***n*** **_Case_Total**		***n*** **_Case_Clinic**	**%_Total**
5	Night game_yes	219		219	41.2
6	Solo game_yes	162		162	30.5
7	Time taken away from study_yes	56		56	10.5
8	Time taken away from friends_yes	169		169	31.8
9	Time has taken away from going out_yes	35		35	6.6
10	Time taken away from personal hygiene_yes	45		45	8.5
11	Time has taken away from eating_yes	305		271	57.3
12	Reason for addiction: absence of friends_yes	138		138	25.9
13	Reason for addiction: reduced interest in the outdoors_yes	56		56	10.5
14	Reason justifying addiction: poor school performance_yes	33		33	6.2
15	Reason justifying addiction: emotional and family difficulties_yes	443		271	83.3
16	Alterations in life conduction as a result of video game-like activity	286		271	53.8
17	Reinforcement of video game addiction in pandemic_yes	326		271	61.3
18	Use of lies_yes	229		229	43.0
19	Anger_yes	234		234	44.0
20	Aggressiveness_yes	266		266	50.1
21	Subjective perception of benefits from using video games_yes	341		271	64.1
22	Preferred game setting (violence) _yes	449		271	84.4
23	Use of video games in online mode_yes	396		271	74.4
24	Online encounters with individuals younger than the recommended age_yes	417		271	78.4
25	Witnessing others of deviant or criminal conduct_yes	417		271	78.4
26	Simulation or implementation of deviant or criminal conduct_yes	417		271	78.4

**Table 4:** T-test for independent variables (dependent variable: #3).

N_Variable	Nomenclature	F	t	gl	*p*
1	Age	2.152	−0.904	529	0.366
2	Gender	25.347	−5.288	529	**0.000**
4	Time spent per day on recreational activities such as video games	294.708	−19.234	529	**0.000**
5	Night-game_yes	58.634	−7.759	529	**0.000**
6	Solo-game_yes	5.012	1.115	529	0.265
7	Time taken away from study_yes	42.740	3.293	529	**0.001**
8	Time taken away from friends_yes	2.797	0.834	529	0.405
9	Time has taken away from going out_yes	9.290	−1.538	529	0.125
10	Time taken away from personal hygiene_yes	8.520	1.448	529	0.148
11	Time has taken away from eating_yes	3.595	0.945	529	0.345
12	Reason for addiction: absence of friends_yes	0.089	0.149	529	0.882
13	Reason for addiction: reduced interest in the outdoors_yes	4.603	−1.074	529	0.283
14	Reason justifying addiction: poor school performance_yes	1.873	0.683	529	0.495
15	Reason justifying addiction: emotional and family difficulties_yes	2.415	0.775	529	0.439
16	Alterations in life conduction as a result of video game-like activity	4.558	−1.063	529	0.288
17	Reinforcement of video game addiction in pandemic_yes	8.169	−2.350	529	**0.019**
18	Use of lies_yes	27.039	−2.843	529	**0.005**
19	Anger_yes	13.131	−2.174	529	**0.030**
20	Aggressiveness_yes	11.240	−2.089	529	**0.037**
21	Subjective perception of benefits from using video games_yes	0.022	−1.521	529	0.129
22	Preferred game setting (violence) _yes	3.951	−0.998	529	0.319
23	Use of video games in online mode_yes	13.675	−1.829	529	0.068
24	Online encounters with individuals younger than the recommended age_yes	1.061	−0.515	529	0.607
25	Witnessing others of deviant or criminal conduct_yes	12.486	−1.753	529	0.080
#26	Simulation or implementation of deviant or criminal conduct_yes	12.486	−1.753	529	0.080

**Table 5:** Binary correlations between clinical variables. R: correlation coefficient; *p*: significance.

N	Variable	Age (1)		Gender (2)		Cluster (3)	
		R	*p*	R	*p*	R	*p*
4	Time spent per day on recreational activities such as video games	0.151	**0.000**	0.210	**0.000**	0.934	**0.000**
5	Night game_yes	0.102	**0.018**	0.103	**0.018**	0.320	**0.000**
6	Solo game_yes	0.117	**0.007**	0.122	**0.005**	0.048	0.265
7	Time taken away from study_yes	0.043	0.317	−0.016	0.705	0.142	**0.001**
8	Time taken away from friends_yes	0.168	**0.000**	−0.007	0.874	0.036	0.405
9	Time has taken away from going out_yes	−0.048	0.268	0.009	0.835	−0.067	0.125
10	Time taken away from personal hygiene_yes	−0.060	0.167	−0.022	0.612	0.063	0.148
11	Time has taken away from eating_yes	0.044	0.309	−0.014	0.754	0.041	0.345
12	Reason for addiction: absence of friends_yes	0.171	**0.000**	0.085	**0.049**	0.006	0.882
13	Reason for addiction: reduced interest in the outdoors_yes	0.180	**0.000**	−0.075	0.085	−0.047	0.283
14	Reason justifying addiction: poor school performance_yes	0.092	**0.035**	0.048	0.270	0.030	0.495
15	Reason justifying addiction: emotional and family difficulties_yes	0.093	**0.031**	0.099	**0.022**	0.034	0.439
16	Alterations in life conduction as a result of video game-like activity	−0.015	0.731	0.025	0.568	−0.046	0.288
17	Reinforcement of video game addiction in pandemic_yes	0.198	**0.000**	−0.010	0.816	0.102	**0.019**
18	Use of lies_yes	0.575	**0.000**	0.089	**0.040**	0.123	**0.005**
19	Anger_yes	0.054	0.218	0.033	0.445	0.094	**0.030**
20	Aggressiveness_yes	0.055	0.206	0.027	0.532	0.090	**0.037**
21	Subjective perception of benefits from using video games_yes	0.087	**0.045**	0.123	**0.005**	−0.066	0.129
22	Preferred game setting (violence) _yes	0.035	0.418	−0.047	0.285	−0.043	0.319
23	Use of video games in online mode_yes	−0.031	0.477	0.199	**0.000**	−0.079	0.068
24	Online encounters with individuals younger than the recommended age_yes	0.082	0.060	0.014	0.756	−0.022	0.607
25	Witnessing others of deviant or criminal conduct_yes	0.233	**0.000**	0.045	0.301	−0.076	0.080
26	Simulation or implementation of deviant or criminal conduct_yes	0.263	**0.000**	0.045	0.301	−0.076	0.080

## Data Availability

The data were received and stored by Giovanni Della Porta.
